# TERT Promoter Mutations Differently Correlate with the Clinical Outcome of MAPK Inhibitor-Treated Melanoma Patients

**DOI:** 10.3390/cancers12040946

**Published:** 2020-04-11

**Authors:** Paola Del Bianco, Camilla Stagni, Silvia Giunco, Alessio Fabozzi, Lisa Elefanti, Stefania Pellegrini, Antonella Vecchiato, Jacopo Pigozzo, Carolina Zamuner, Anita De Rossi, Arcangela De Nicolo, Chiara Menin

**Affiliations:** 1 Clinical Research Unit, Veneto Institute of Oncology IOV – IRCCS, 35128 Padua, Italy; paola.delbianco@iov.veneto.it; 2 Immunology and Diagnostic Molecular Oncology Unit, Veneto Institute of Oncology IOV – IRCCS, 35128 Padua, Italy; camilla.stagni3@gmail.com (C.S.); lisa.elefanti@iov.veneto.it (L.E.); anita.derossi@unipd.it (A.D.R.); 3 Department of Molecular Medicine, University of Padua, 35128 Padua, Italy; 4 Oncology and Immunology Section, Department of Surgery, Oncology and Gastroenterology, University of Padua, 35128 Padua, Italy; silvia.giunco@unipd.it (S.G.); stefania.pellegrini.1@phd.unipd.it (S.P.); 5 Veneto Institute of Oncology IOV – IRCCS, 35128 Padua, Italy; alessio.fabozzi@iov.veneto.it; 6 Surgical Oncology Unit, Veneto Institute of Oncology IOV – IRCCS, 35128 Padua, Italy; antonella.vecchiato@iov.veneto.it; 7 Melanoma Oncology Unit, Veneto Institute of Oncology IOV – IRCCS, 35128 Padua, Italy; jacopo.pigozzo@iov.veneto.it; 8 Anatomy and Histology Unit, Veneto Institute of Oncology IOV – IRCCS, 35128 Padua, Italy; carolina.zamuner@iov.veneto.it; 9 Cancer Genomics Program, Veneto Institute of Oncology IOV – IRCCS, 35128 Padua, Italy; arcangela.denicolo@iov.veneto.it

**Keywords:** melanoma, TERT promoter, MAPK pathway, MAPK inhibitors

## Abstract

Resistance is a major challenge in the management of mitogen-activated protein kinase inhibitor (MAPKi)-treated metastatic melanoma. Tumor genetic alterations can cause MAPK pathway reactivation, leading to lack of response and poor outcome. Characterization of the mutational profile in patients with melanoma might be crucial for patient-tailored treatment choices. Mutations in the promoter region of the telomerase reverse transcriptase gene (TERTprom) lead to increased TERT expression and telomerase activity and are frequent in BRAF^V600^ mutant melanoma. Reportedly, TERTprom, and BRAF^V600^ mutations cooperate in driving cancer progression and aggressiveness. We evaluated the effect of the TERTprom status on the clinical outcome in 97 MAPKi-treated melanoma patients. We observed that patients with the c.-146C > T mutation showed a significantly worse progression-free survival (PFS) compared to those carrying the c.-124C > T mutation and a two-fold increased risk of progression (median 5.4 vs. 9.5 months; hazard ratio (HR) 1.9; 95% confidence interval (CI) 1.2–3.2; *p* = 0.013). This trend was also observed for the overall survival (OS); melanoma patients with the c.-146C > T mutation showed a poorer prognosis compared to those with the c.-124C > T mutation (median 13.3 vs. 25.5 months; HR 1.9, 95% CI 1.1–3.3, *p =* 0.023). Our results disclose a different correlation of the two TERTprom mutations with MAPKi-treated melanoma patient outcome, highlighting a different impact of the pathway blockade.

## 1. Introduction 

The advent of the mitogen-activated protein kinase inhibitor (MAPKi) significantly improved the management of patients with BRAF^V600^-mutated metastatic melanoma [1]. Nevertheless, drug resistance invariably develops in the majority of the patients receiving MAPKi. Several reports showed that more than half of the resistance mechanisms to targeted therapy are due to genomic alterations, already present at baseline, which cause reactivation of the MAPK pathway [1,2]. Thus, definition of the mutational profile of the melanoma before treatment initiation could be useful to identify the patients who might benefit from targeted therapy. Metastatic melanoma is one of the cancers with the highest mutational burden. Mutations in the promoter region of the telomerase reverse transcriptase gene (TERTprom), along with mutations in BRAF/NRAS, are the most frequent genetic alterations detected in melanoma [3]. While the prognostic significance of BRAF/NRAS mutations in melanoma is still debated, numerous studies reported that TERTprom mutations associate with poor prognosis [4,5,6,7]. TERT encodes the catalytic subunit of the telomerase complex, which plays a key role in maintaining chromosomal telomere length, thus supporting cell survival. The two most frequent, mutually exclusive, TERTprom mutations map at 146 and 124 base pairs (bp) upstream of the translational start site of TERT (c.-146C > T and c.-124C > T) and generate an identical binding site for the E-twenty six/ternary complex factors (Ets/TCF) which activate TERT expression [8]. A link between the BRAF^V600^-activated MAPK pathway and TERTprom mutation function was recently described, whereby the BRAF^V600^-activated MAPK pathway, via FOS stabilization, would promote formation of a GA binding protein (GABP) transcriptional complex that in turn would bind TERTprom mutations, thus upregulating TERT expression [9]. In addition, unlike the c.-124C > T mutation, the c.-146C > T can activate TERT transcription via an alternative mechanism by binding with the non-canonical NF-κB (nuclear factor kappa-light-chain-enhancer of activated B cells) signaling-induced p52/ETS complex [10]. The effect of the TERTprom mutations on TERT expression may be further complicated by the presence of the rs2853669 single nucleotide polymorphism (SNP), which reportedly disrupts a pre-existing ETS binding site at −245 bp in the TERTprom region and, hence, counteracts the transactivation activity of the TERTprom hotspots [11]. However, the effect of this SNP on prognosis is still controversial. In glioblastoma, poor survival was described in TERTprom mutated patients who did not carry the SNP [12,13], as well as in those who were homozygous for the minor allele [14,15]. In liver cancer patients, the combination of the TERT mutations with the SNP reportedly increased the risk of recurrence and the mortality [16]. In melanoma patients, the negative prognostic effect of the TERTprom mutations was maintained only in patients who did not carry the rs2853669 SNP, which, per se, was reported to be associated with longer survival [4].

We set out to evaluate if the TERTprom status correlates with telomere length and clinical outcome in a cohort of BRAF^V600^-mutated melanoma patients who received MAPKi therapy. Our findings suggest that the association of the TERTprom status with prognosis is complex and different depending on the mutation.

## 2. Results

### 2.1. Clinical Characteristics 

A total of 97 patients (40 females and 57 males, overall median age 55 years) with advanced melanoma treated with BRAF inhibitors in monotherapy (41.2%) or in combination with MEK inhibitors (58.8%) were included in the analysis. At the beginning of treatment, 85 patients presented a stage IV disease (28.2% with cerebral metastases), 11 presented a stage IIIC disease, and one presented a stage IIIB disease, based on the eighth edition of the American Joint Committee on Cancer staging system. Performance status (PS) according to the European Cooperative Oncology Group (ECOG) scale was 0 in 70.1% of cases. Highly elevated serum lactate dehydrogenase (LDH) at baseline (>2× upper limit of normal, ULN) was observed in 14 out of the 84 patients with available data, while 19 and 51 patients had modestly elevated (>ULN - ≤2ULN) and normal (≤ULN) serum LDH, respectively. Two patients died during treatment, while 83 progressed on therapy (15 out of these were still alive at the time of the analysis): two patients continued beyond progression, 54 patients were treated with subsequent lines of therapy, one patient underwent surgery without further treatment, and 26 patients received only best supportive care. In particular, after MAPKi, 18 patients received only immunotherapy with cytotoxic T lymphocyte-associated protein 4 (CTLA-4) and/or programmed cell death-1 (PD-1) inhibitors, while 27 received only chemotherapy with temozolomide or carboplatin + paclitaxel. Seven patients received more than one line: two patients received immunotherapy followed by chemotherapy; one received chemotherapy followed by immunotherapy; one received cyclin dependent kinase (CDK) inhibitor-based treatment followed by immunotherapy; two received two subsequent lines of chemotherapy; one received three lines of treatment, including immunotherapy, chemotherapy, and targeted therapy. Two patients were treated elsewhere, and no subsequent therapeutic details were available. Among the 12 patients without progression, 10 were still on treatment and two stopped treatment for toxicity after 54 months and 44 months. The overall progression-free survival (PFS) was 6.9 months (95% confidence interval (CI), 5.5–8.7), specifically, 5.4 months (95% CI, 4.1–6.4) for patients treated with monotherapy and 8.7 months (95% CI, 6.6–15.6) for those who received combo therapy. The median overall survival (OS) was 15.3 months (95% CI, 10.3–23.9). Patient demographics and clinical features are summarized in Table 1 and detailed in Table S1 (Supplementary Materials).

### 2.2. TERT Promoter Status and Telomere Length 

TERTprom mutational status was evaluated in pre-treatment tumor specimens. Consistent with previous reports, we detected a high frequency (79.4%) of TERTprom mutations in our cohort of BRAF^V600^ melanoma patients. The most common mutations were the c.-124C > T, the c.-146C > T (*n* = 36, each), and the c.-138/-139CC > TT (*n* = 5) mutations (Table 1). The three mutations were mutually exclusive. Seven patients showed a second mutation in the TERTprom region (Table S1, Supplementary Materials). We also genotyped our cohort of patients for the rs2853669 SNP at −245 bp. A total of 52 patients (53.6%) carried the (minor) C-variant allele (Table 1), for which 10 patients were homozygous and 42 were heterozygous (Table S1, Supplementary Materials). Notably, we observed a different distribution of TERTprom mutations according to the age at therapy initiation (corresponding to the age at diagnosis of the metastatic disease). Specifically, patients with the c.-124C > T mutation had a significantly lower median age (*p* = 0.002) (Table 1).

We then measured the relative telomere length (TL, see Section 4 for details) in 80 pre-treatment melanoma samples (Table S1, Supplementary Materials). Values ranged between 0.5 and 5.1 (median 1.5) and did not correlate with the age (data not shown). In addition, median levels of telomere length did not significantly change according to TERTprom mutations (Table 1) or any other investigated variable (Table S2, Supplementary Materials). Median and interquartile TL values, stratified according to patients’ clinical characteristics, are shown in Table S2 (Supplementary Materials).

### 2.3. Association with Progression-Free Survival

We investigated the association between TERTprom status at baseline and PFS after MAPKi treatment. In univariate analysis, no difference was observed between patients whose tumors carried any TERTprom mutation and the TERT wild-type (wt) ones (median PFS 6.9 months vs. 7.2 months; hazard ratio (HR) 1.1, 95% CI, 0.7–1.9, *p = 0.66*) (Table 2). However, upon stratification based on mutation type, patients carrying the c.-146C > T mutation displayed significantly shorter PFS compared to those with the c.-124C > T with a two-fold increased risk of progression (median PFS 5.4 months vs. 9.5 months; HR 1.9, 95% CI, 1.2–3.2, *p* = *0.013*) (Table 2; Figure S1, Supplementary Materials). A significant difference was still appreciated when the seven patients, whose melanoma displayed a double TERTprom mutation, were excluded from the analysis (data not shown). Furthermore, age, gender, ECOG PS, serum LDH, presence of brain metastasis, and type of therapy were significantly correlated to PFS in the univariate analysis, whereas the rs2853669 polymorphism (specifically, its C-variant) and telomere length were not. Additionally, we did not detect significant interaction between TERTprom mutations and rs2853669 SNP (data not shown). 

**Figure 1:**
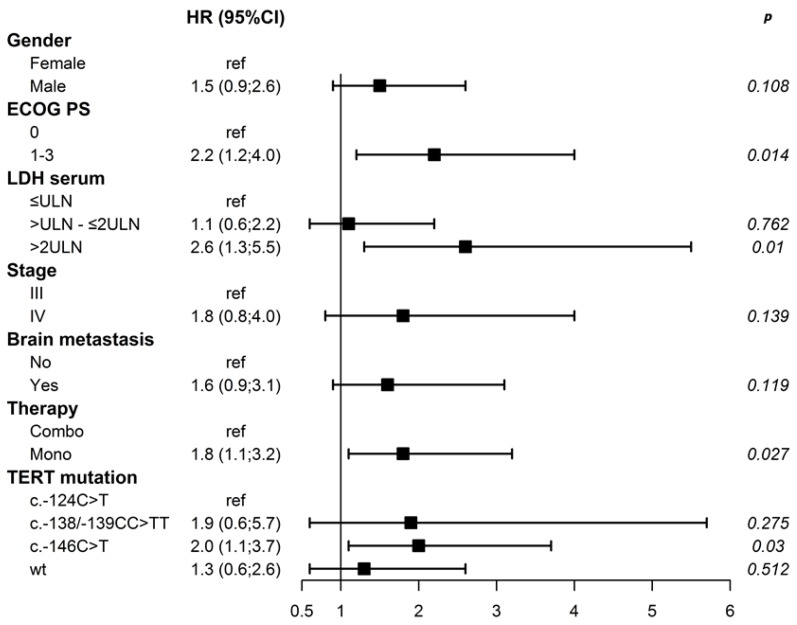
Forest plot showing hazard ratios for progression-free survival using a multivariable Cox proportional hazards model.

In the multivariable model, ECOG PS, serum LDH, type of therapy, and TERTprom mutation remained as independent predictors of PFS, and the c.-146C > T genotype showed a double risk of progression compared to the c.-124C > T genotype (HR 2.0, 95% CI, 1.1–3.7, *p* = 0.03) (Figure 1).

### 2.4. Association with Overall Survival

**Figure 2:**
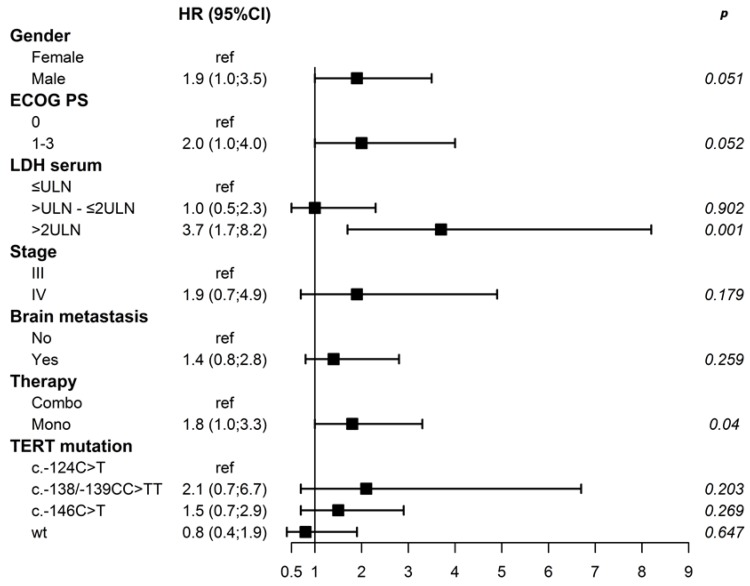
Forest plot showing hazard ratios for overall survival using a multivariable Cox proportional hazards model.

When the effects of several variables, including TERTprom status, were evaluated with respect to the OS, we observed that age, gender, ECOG PS, serum LDH, stage, presence of brain metastasis, and type of therapy were significantly correlated to the OS (Table 3). In addition, as for PFS, in univariate analysis, melanoma patients with the c.-146C > T TERTprom mutation showed significantly shorter OS compared to those with the c.-124C > T mutation (median 13.3 months vs. 25.5 months) with HR 1.9, 95% CI 1.1–3.3, *p = 0.023* (Table 3; Figure S2, Supplementary Materials). The shortest, albeit non-significant, OS (8.2 months) was observed in the few c.-138/-139CC > TT patients. Notably, in multivariable analysis, only serum LDH and therapy remained significantly associated with OS (Figure 2).

## 3. Discussion 

Mutations in the TERTprom region occur frequently in BRAF^V600^-mutant melanoma [17] and were outlined as markers of tumor aggressiveness and poor prognosis [4,18]. Reportedly, the two most frequent TERTprom mutations, c.-124C > T and c.-146C > T, cooperate with the BRAF^V600^-activated MAPK pathway to promote oncogenesis [9,19,20]. In this study, we evaluated the effect of the two TERT hotspots on the outcome of melanoma patients treated with MAPKi. In our melanoma samples, we observed that the two mutations had the same frequency (37%), and patients with a c.-124C > T mutant tumor showed a significantly younger median age. 

We did not find a different clinical outcome between TERTprom mutated and wt melanoma patients. However, after patient stratification based on TERT mutation type, the c.-124C > T and c.-146C > T mutations differently correlated with patient PFS (9.5 vs. 5.4 months, respectively) and the c.-146C > T mutation associated with a two-fold increased risk of progression compared to the c.-124C > T mutation, suggesting a different role of the two TERTprom mutations on the MAPK pathway blockade. Although both the c.-124C > T and the c.-146C > T were shown to increase transcription of the TERT gene (hence, telomerase activity) by creating new binding sites for ETS transcription factors, previous reports demonstrated that these mutations are functionally distinct. Indeed, a peculiar pathway of activation by non-canonical NF-κB signaling was only described for the c.-146C > T mutation [10,21]. Thus, conceivably, the MAPKi act by inhibiting GABP complex transactivation on both TERT mutations, while the non-canonical NF-κB signaling, activated by cytokines and receptors of the tumor necrosis factor receptor (TNFR) superfamily [22], promotes p52/ETS1 binding to the c.-146C > T mutation only, possibly contributing to the worse PFS in these subgroup of MAPKi-treated melanoma patients. 

When we performed overall survival analysis, the TERT c.-146C > T mutation still correlated with worse outcome compared to the c.-124C > T mutation, although significance was lost after adjusting for the other clinical variables. We acknowledge that the OS might be confounded by the subsequent treatments after MAPKi, which were highly heterogeneous and were not included in the statistical analysis. 

In line with a study in a stage I/II melanoma cohort [6], we observed that, when patients were stratified according to TERTprom mutations, those with c.-138/-139CC > TT mutated melanoma had the shortest survival (although not statistically significant, probably owing to the small number of cases). The remainder of our results are not aligned, however, with the data by Andres-Lencina et al. who reported that the c.-146C > T mutation had the least pronounced effect on survival. Notably, their data mostly refer to untreated patients with stage I/II melanoma, whereas our study specifically focused on metastatic melanoma patients after MAPKi therapy. Moreover, Andres-Lencina et al. and TCGA studies [6,23] reported a higher TERTprom activity and TERT expression levels in untreated patients with c.-124C > T mutated melanoma compared to those with other TERT mutations. Nevertheless, in melanoma cell lines, a dramatic decline in TERT transcription and telomerase activity was observed after short-term exposure to MAPKi, regardless of TERTprom mutation [20]. We hypothesize that the aforementioned non-canonical NF-κB signaling pathway, which, in vivo, is activated by microenvironment factors, might not be functional in the -146C > T mutated cell lines, thus not allowing the overcoming of the MAPK blockade. 

We report a lack of association between TERTprom mutations and telomere length in our pre-treatment melanoma specimens even after stratification based on mutation type. Moreover, the telomere length was not associated with PFS in our cohort of MAPKi-treated patients. Conceivably, the expected increase of TERTprom mutation-driven telomerase activity might not suffice to counteract telomere shortening during advanced disease [24]. Presumably, this scenario might be maintained after the MAPK pathway block by the activation of non-canonical NF-κB signaling only in tumors with c.-146C > T TERTprom. Future in vitro studies should clarify if BRAF/TERT mutant melanoma cell lines, after exposure to MAPKi and to stimuli activating the TNFR superfamily, show different TERT expression and telomerase activity, as well as different telomere length based on TERTprom mutation type.

When we investigated the role of the rs2853669 polymorphism at −245 bp, which reportedly counteracts the activating effect of the above-mentioned TERTprom mutations [25] and modulates their negative effect on melanoma survival [4], we found that the SNP did not interfere with the effect of the two hotspots on treatment outcome and was not associated with telomere length, as opposed to what was observed in melanoma cell lines [26]. 

Our results showed that the two most common TERT mutations were differently associated with PFS on MAPKi treatment, supporting both a functional link between TERT biology and the MAPK pathway and uncovering different behaviors of the c.-146C > T and c.-124C > T TERT mutations. 

## 4. Patients and Methods 

### 4.1. Patient Cohort

A total of 97 specimens were collected, prior to MAPK-targeted therapy initiation, from 97 patients diagnosed with BRAF^V600^ mutant unresectable stage III or stage IV cutaneous melanoma, who were treated at the Veneto Institute of Oncology IRCCS in Padua between October 2011 and January 2020.

All patients received MAPK inhibitor therapy; 40 (41.2%) were treated with monotherapy (vemurafenib or dabrafenib), and 57 (58.8%) were treated with a combination of BRAF and MEK inhibitors (dabrafenib + trametinib, vemurafenib + cobimetinib). We used PFS—calculated from the date of MAPKi treatment initiation to the date of the documented progression or death—as a clinical outcome measure. Overall tumor burden was measured by total body computed tomography (CT) scan, including brain, with contrast at baseline and then every three cycles. Clinical response was assessed according to Response Evaluation Criteria in Solid Tumors (RECIST) version 1.1. Information about age, gender, clinical history including stage, presence of brain metastasis, serum LDH, therapy, and ECOG PS at baseline was collected. As a secondary outcome measure, we estimated OS, defined as the time from MAPKi treatment initiation to the patient’s death by any cause.

Written informed consent was obtained from all patients before enrollment in the study, which was approved by the Ethics Committee of the Veneto Institute of Oncology IRCCS (Cod. Int. CE IOV: 2015/85) and carried out in accordance with the principles of the Declaration of Helsinki. 

### 4.2. Tumor Samples and DNA Extraction

All melanoma specimens were formalin-fixed paraffin-embedded (FFPE). A pathologist contoured and estimated the tumor area on hematoxylin and eosin-stained slides. Samples with tumor content ≥50% were macrodissected to enrich the tumor cell population. DNA was extracted using the QIAmp DNA micro/mini kit (Qiagen, Hilden, Germany) or the MagNA Pure Compact Instrument (Roche Diagnostics, Mannheim, Germany) according to the manufacturer’s instructions.

### 4.3. End-Point PCR and Sanger Sequencing

The mutational status of the TERTprom region (from −27 to −286 upstream of the ATG), including the rs2853669 polymorphic site, was assessed by PCR amplification and Sanger sequencing. PCR was carried out in a 50 µL volume containing 100 ng of DNA, 5 µL of AmpliTaq Gold 10× buffer, 0.2 mM dNTPs, 1 mM MgCl_2_, 0.5 µM of each M13-tailed primer, 10 µL of GC enhancer for sample to amplify, and 1.25 U of AmpliTaq Gold 360 polymerase. The AmpliTaq Gold 360 reagents were purchased from Applied Biosystems (Austin, TX, USA). Primer sequences were previously reported by Heidenreich et al. [17]. The amplified products were purified with the Illustra GFX 96 PCR Purification Kit (GE Healthcare, Buckinghamshire, UK) and sequenced using Big Dye terminator v1.1 Cycle Sequencing Kit (Applied Biosystems, Austin, TX, USA) and Big Dye XTerminator Purification Kit (Thermo Fisher Scientific, Waltham, MA, USA) on a 96-capillary sequencer (AB3730xl Genetic Analyzer).

### 4.4. Telomere Length

Telomere length was determined by multiplex PCR as previously described [27,28]. Briefly, each PCR reaction was performed in a final volume of 25 μL, containing 5 μL of sample (10 ng of DNA) and 20 μL of reaction mix containing 0.75× SYBR GreenI (Invitrogen, Carlsband, CA, USA), 10 mmol/L Tris-hydrochloric acid pH 8.3, 50 mmol/L potassium chloride, 3 mmol/L magnesium chloride, 0.2 mmol/L each deoxynucleotide (dNTP) (Applied Biosystems, Foster City, California, USA), 1 mmol/L dithiothreitol, 0.625 U AmpliTaq Gold DNA polymerase (Applied Biosystems), 1% dimethyl sulfoxide (Sigma-Aldrich, St Louis, Missouri, USA), and 900 nmol/L of each of the primers. PCR reactions were performed on a LightCycler480 real-time PCR detection system (Roche Applied Science, Mannheim, Germany). A standard curve was generated at each PCR run, consisting of DNA from the RAJI cell line, serially diluted from 20 to 0.25 ng/µL [29]. All DNA samples and reference samples were run in triplicate. The LC480Conversion and the LinRegPCR free softwares were used to converted raw text files and to analyze the converted data. Telomere length (TL) values were calculated as telomere/single-copy gene (T/S) ratio, as previously described [29]. The intra- and inter-assay variability of the TL values were evaluated using reference samples; coefficients of variation were 3.95% (or less) and 6.05% (or less), respectively. In 17 cases, TL was not evaluable due to the insufficient amount of DNA. 

### 4.5. Statistical Analysis 

Age and TL were considered as continuous variables and summarized as median and range. Categorical variables were reported as counts and percentages. The association of patients’ characteristics with the TERTprom mutation was assessed using the Mann–Whitney test and the χ^2^ or Fisher exact test as appropriate. The impact of patients’ characteristics on TL was estimated by robust linear regression adjusted for age, and a robust F-test was used for significance. PFS and OS probabilities were computed using the Kaplan–Meier method. Patients who did not develop an event during the study period were censored at the date of last observation. The median survival times and 95% CI were reported. HRs and 95% CI for each group were estimated using univariate Cox proportional hazards models with low risk as the reference class. The independent role of each covariate in predicting survival was verified in a multivariable model considering all characteristics significantly associated with the outcome in the univariate analyses. All tests were two-sided, and a *p*-value <0.05 was considered statistically significant. Statistical analyses were performed using the SAS version 9.4 (SAS Institute, Cary, NC, USA) and the RStudio (RStudio: Integrated Development for R. RStudio, Inc., Boston, MA).

## 5. Conclusions

We found that the TERTprom mutations differently correlate with the clinical outcome of MAPKi-treated melanoma patients. Our study, although not ascertaining a predictive role of the TERTprom mutations, highlights the complexity of the pathways that underlie resistance to targeted therapy and regulate TERT expression and its impact on the prognosis in melanoma.

## Figures and Tables

**Table 1:** Demographics, clinical and molecular variables, and their associations with telomerase reverse transcriptase (TERT) promoter mutations. ECOG PS—European Cooperative Oncology Group Performance Status; LDH—lactate dehydrogenase; ULN—upper limit of normal; SNP—single nucleotide polymorphism; TL—telomere length; wt—wild type.

Characteristics			TERT Promoter Mutation				
		All Cases *N* = 97 (%)	c.-124C > T *N* = 36 (37.1%)	c.-138/-139CC > TT *N* = 5 (5.2%)	c.-146C > T *N* = 36 (37.1%)	wt *N* = 20 (20.6%)	*p*
Age (years)	Median (Q1; Q3)	55 (46; 67)	49.5 (38.7; 60.2)	66 (62; 71)	62.5 (49.7; 70)	54 (44.7; 61.7)	0.002
Gender	Female	40 (41.2%)	18 (50.0%)	2 (40.0%)	12 (33.3%)	8 (40.0%)	0.5371
	Male	57 (58.8%)	18 (50.0%)	3 (60.0%)	24 (66.7%)	12 (60.0%)	
ECOG PS	0	68 (70.1%)	23 (63.9%)	4 (80.0%)	28 (77.8%)	13 (65.0%)	0.5732
	1–3	29 (29.9%)	13 (36.1%)	1 (20.0%)	8 (22.2%)	7 (35.0%)	
Serum LDH	≤ULN	51 (60.7%)	20 (58.8%)	3 (60.0%)	19 (67.9%)	9 (52.9%)	0.8904
	>ULN to ≤2ULN	19 (22.6%)	9 (26.5%)	1 (20.0%)	4 (14.3%)	5 (29.4%)	
	>2ULN	14 (16.7%)	5 (14.7%)	1 (20.0%)	5 (17.9%)	3 (17.6%)	
	Unknown	13					
Stage	III	12 (12.4%)	6 (16.7%)	0	3 (8.3%)	3 (15.0%)	0.7194
	IV	85 (87.6%)	30 (83.3%)	5	33 (91.7%)	17 (85.0%)	
Brain metastasis	No	71 (74.7%)	29 (80.6%)	3 (60.0%)	27 (79.4%)	12 (60.0%)	0.2407
	Yes	24 (25.3%)	7 (19.4%)	2 (40.0%)	7 (20.6%)	8 (40.0%)	
	Unknown	2					
Therapy	Combo	57 (58.8%)	22 (61.1%)	2 (40.0%)	20 (55.6%)	13 (65.0%)	0.7445
	Mono	40 (41.2%)	14 (38.9%)	3 (60.0%)	16 (44.4%)	7 (35.0%)	
SNP rs2853669	T/T	45 (46.4%)	17 (47.2%)	1 (20.0%)	19 (52.8%)	8 (40.0%)	0.5417
	C/C-C/T	52 (53.6%)	19 (52.8%)	4 (80.0%)	17 (47.2%)	12 (60.0%)	
TL (*N* = 80)	Median (Q1; Q3)	1.5 (1.2; 1.9)	1.6 (1.2; 1.8)	1.8 (1.6; 2.1)	1.4 (1.2; 1.7)	1.4 (1.2; 2.0)	0.5301 * 0.7330 ^

* Adjusted and ^ unadjusted for age.

**Table 2:** Univariate Cox proportional hazards models of progression free survival. CI—confidence interval; HR—hazard ratio; NE—not estimable.

Characteristics		Events	Median (95% CI)	HR	95% CI	*p*
Age (years)				1.02	1.00; 1.04	0.0167
Gender	Female	34/40	9.7 (6.6; 17.3)	Reference		
	Male	51/57	5.4 (4.7; 7.0)	2.0	1.2; 3.1	0.0044
ECOG PS	0	59/68	9.7 (6.9; 14.5)	Reference		
	1–3	26/29	5.3 (2.7; 5.5)	2.0	1.2; 3.2	0.0042
Serum LDH	≤ULN	42/51	11.4 (6.5; 15.4)	Reference		
	>ULN to ≤2ULN	16/19	6.5 (5.4; 10.3)	1.2	0.7; 2.2	0.5193
	>2ULN	14/14	2.9 (2.0; 5.3)	3.2	1.7; 6.0	<0.0001
Stage	III	9/12	19.6 (6.5; 53.3)	Reference		
	IV	76/85	6.4 (5.4; 7.4)	2.0	1.0; 4.0	0.0537
Brain metastasis	No	59/71	8.1 (6.3; 14.5)	Reference		
	Yes	24/24	5.3 (3.1; 6.5)	2.4	1.5; 4.0	<0.0001
Therapy	Combo	45/57	8.7 (6.6; 15.6	Reference		
	Mono	40/40	5.4 (4.1; 6.4)	2.1	1.4; 3.3	<0.0001
TERT status	wt	17/20	7.2 (3.5; 15.6)	Reference		
	Mutated	68/77	6.9 (5.4; 9.1)	1.1	0.7; 1.9	0.6592
TERT mutation	c.-124C > T	30/36	9.5 (5.5; 17.3)	Reference		
	c.-138/-139CC > TT	4/5	6.3 (0.4; NE)	1.7	0.6; 4.9	0.3280
	c.-146C > T	34/36	5.4 (4.1; 8.1)	1.9	1.2; 3.2	0.0130
	wt	17/20	7.2 (3.5; 15.6)	1.2	0.7; 2.2	0.5438
SNP rs2853669	T/T	39/45	7.6 (5.5; 15.0)	Reference		
	C/C-C/T	46/52	5.9 (5.2; 7.4)	1.3	0.8; 1.9	0.2851
TL				1.0 1.1	0.8; 1.4 0.8; 1.5	0.9305 * 0.4633 ^

* Adjusted and ^ unadjusted for age.

**Table 3:** Univariate Cox proportional hazards models of overall survival.

Characteristics		Events	Median (95% CI)	HR	95% CI	*p*
Age (years)				1.02	1.00; 1.04	0.0371
Gender	Female	26/40	25.5 (13.3; 58.0)	Reference		
	Male	44/57	10.3 (7.6; 17.6)	2.3	1.3; 3.8	0.0019
ECOG PS	0	45/68	19.8 (11.5; 39.8)	Reference		
	1–3	25/29	9.5 (5.4; 15.3)	2.3	1.4; 3.7	0.0014
Serum LDH	≤ULN	32/51	21.6 (11.5; 39.1)	Reference		
	ULN to ≤2ULN	14/19	15.3 (7.7; 49.7)	1.2	0.7; 2.3	0.4993
	>2ULN	13/14	5.5 (2.9; 8.2)	3.7	1.9; 7.0	<0.0001
Stage	III	6/12	28.1 (10.9; NE)	Reference		
	IV	64/85	13.3 (8.5; 18.7)	2.3	1.0; 5.4	0.0497
Brain metastasis	No	47/71	21.6 (11.5; 39.1)	Reference		
	Yes	21/24	8.2 (5.4; 15.3)	2.1	1.3; 3.6	0.0040
Therapy	Combo	35/57	23.9 (14.8; 49.7)	Reference		
	Mono	35/40	8.5 (6.6; 12.1)	2.1	1.3; 3.4	0.0014
TERT status	wt	11/20	18.7 (7.2; NE)	Reference		
	Mutated	59/77	14.9 (9.5; 23.9)	1.4	0.7; 2.7	0.3125
TERT mutation	c.-124C > T	24/36	25.5 (10.9; 53.4)	Reference		
	c.-138/-139CC > TT	4/5	8.2 (0.4; NE)	2.4	0.8; 7.0	0.1080
	c.-146C > T	31/36	13.3 (8.0; 18.7)	1.9	1.1; 3.3	0.0230
	wt	11/20	18.7 (7.2; NE)	1.0	0.5; 2.1	0.9906
SNP rs2853669	T/T	31/45	19.8 (10.2; 35.3)	Reference		
	C/C-C/T	39/52	14.8 (8.2; 18.7)	1.4	0.9; 2.3	0.1437
TL (*N* = 80)				1.0 1.1	0.7; 1.4 0.8; 1.5	0.7943 * 0.5241 ^

* Adjusted and ^ unadjusted for age.

## Supplementary Materials

The following are available online at https://www.mdpi.com/2072-6694/12/4/946/s1, Figure S1: Kaplan–Meier curve showing progression-free survival in the patients stratified by TERTprom mutations; Figure S2: Kaplan–Meier curve showing overall survival in the patients stratified by TERTprom mutations; Table S1: Patient demographics, and clinical and molecular features; Table S2: Telomere length distribution within classes of clinical characteristics (test of association adjusted by age).
