# Supplementary material for: TERT Promoter Mutations Differently Correlate with the Clinical Outcome of MAPK Inhibitor-Treated Melanoma Patients
Source: Cancers (Basel). 2020 Apr 11;12(4):946. doi: 10.3390/cancers12040946 (PMC7226422; doi:10.3390/cancers12040946)
Supplement: Supplementary file 1 [file cancers-12-00946-s001.zip › cancers-762003-1rev_Supplementary material/cancers_762003_1rev_Figure S1_S2.pdf]

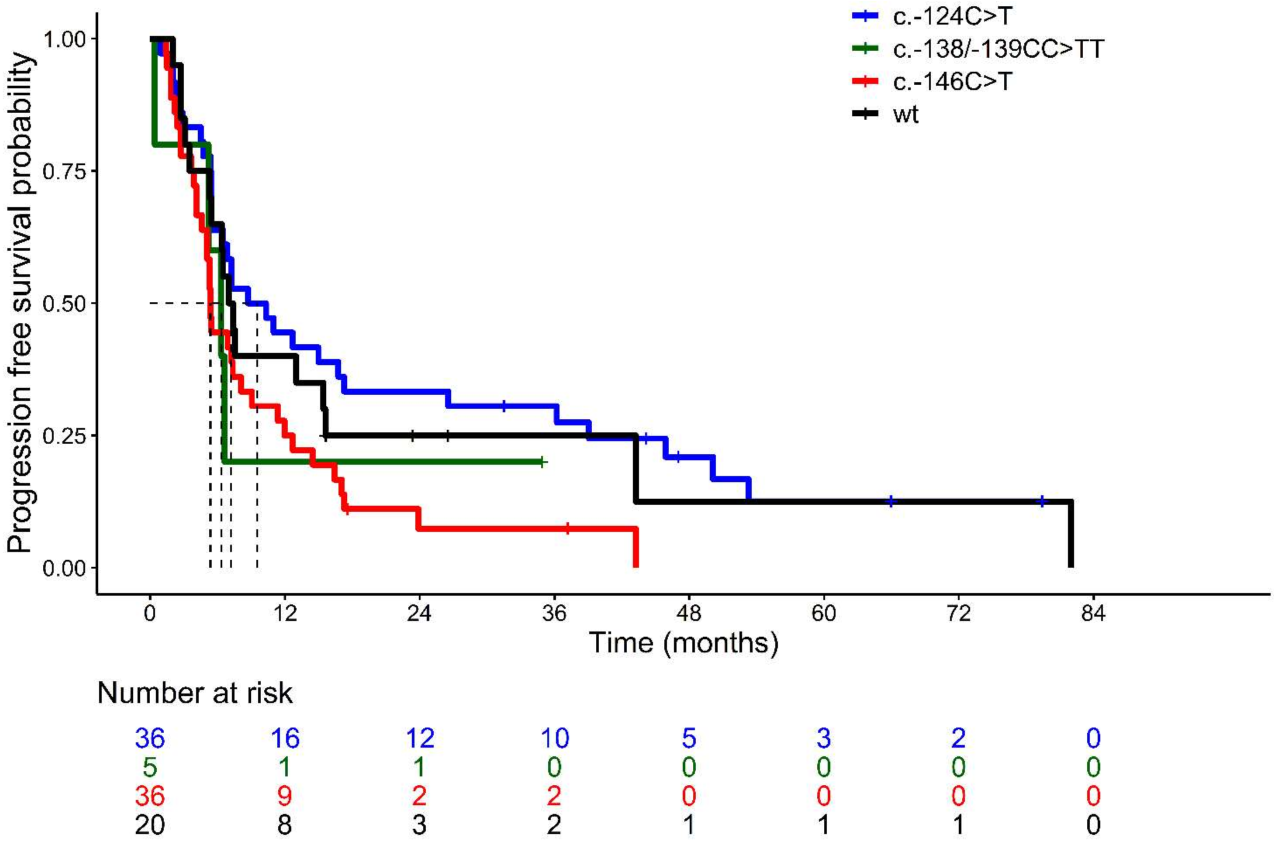

Figure S1. Kaplan-Meier curve showing progression free survival in the patients stratified by TERTprom mutations. The dotted lines reveal the median survival time.

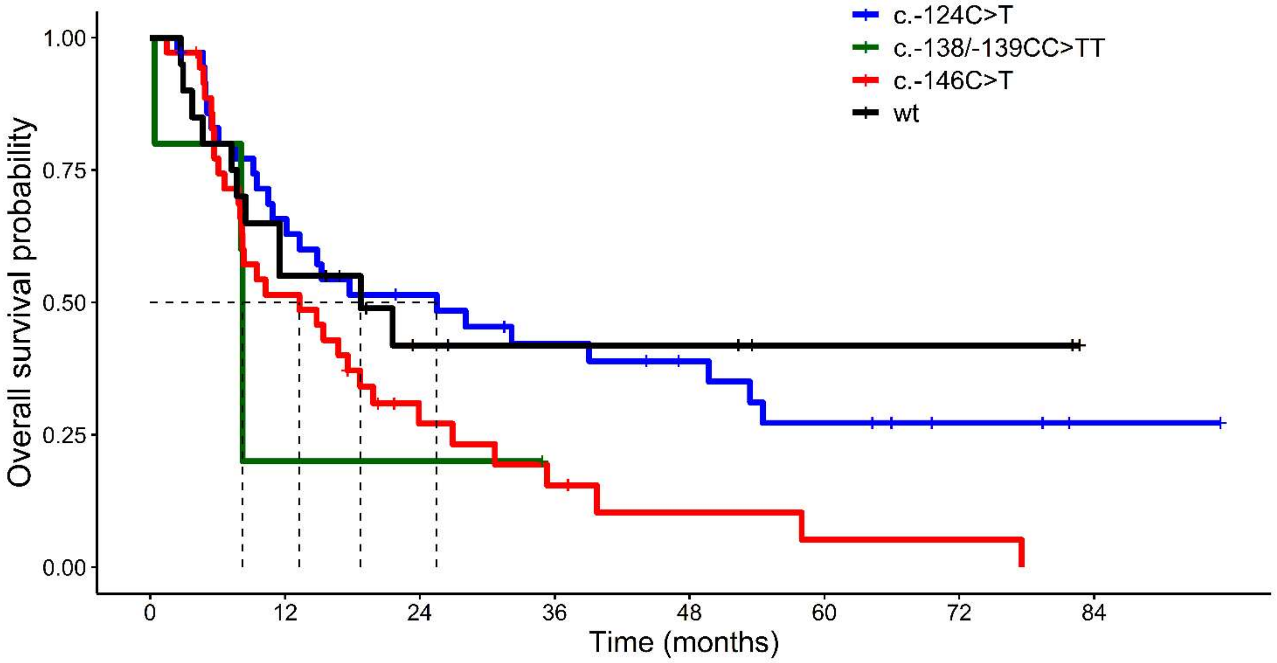

Number at risk

36	23	17	13	10	7	4	1
5	1	1	0	0	0	0	0
36	18	7	4	2	1	1	0
20	11	5	4	4	2	2	0

Figure S2. Kaplan-Meier curve showing overall survival in the patients stratified by TERTprom mutations. The dotted lines reveal the median survival time.
